# Enhancing Medical Student Engagement Through Cinematic Clinical Narratives: Multimodal Generative AI–Based Mixed Methods Study

**DOI:** 10.2196/63865

**Published:** 2025-01-06

**Authors:** Tyler Bland

**Affiliations:** 1 Department of Medical Education, University of Idaho, 875 Perimeter Drive MS 4061, WWAMI Medical EducationMoscow, ID, 83844-9803, United States, 1 5092090908

**Keywords:** artificial intelligence, cinematic clinical narratives, cinemeducation, medical education, narrative learning, AI, medical student, pharmacology, preclinical education, long-term retention, AI tools, GPT-4, image, applicability

## Abstract

**Background:**

Medical students often struggle to engage with and retain complex pharmacology topics during their preclinical education. Traditional teaching methods can lead to passive learning and poor long-term retention of critical concepts.

**Objective:**

This study aims to enhance the teaching of clinical pharmacology in medical school by using a multimodal generative artificial intelligence (genAI) approach to create compelling, cinematic clinical narratives (CCNs).

**Methods:**

We transformed a standard clinical case into an engaging, interactive multimedia experience called “Shattered Slippers.” This CCN used various genAI tools for content creation: GPT-4 for developing the storyline, Leonardo.ai and Stable Diffusion for generating images, Eleven Labs for creating audio narrations, and Suno for composing a theme song. The CCN integrated narrative styles and pop culture references to enhance student engagement. It was applied in teaching first-year medical students about immune system pharmacology. Student responses were assessed through the Situational Interest Survey for Multimedia and examination performance. The target audience comprised first-year medical students (n=40), with 18 responding to the Situational Interest Survey for Multimedia survey (n=18).

**Results:**

The study revealed a marked preference for the genAI-enhanced CCNs over traditional teaching methods. Key findings include the majority of surveyed students preferring the CCN over traditional clinical cases (14/18), as well as high average scores for triggered situational interest (mean 4.58, SD 0.53), maintained interest (mean 4.40, SD 0.53), maintained-feeling interest (mean 4.38, SD 0.51), and maintained-value interest (mean 4.42, SD 0.54). Students achieved an average score of 88% on examination questions related to the CCN material, indicating successful learning and retention. Qualitative feedback highlighted increased engagement, improved recall, and appreciation for the narrative style and pop culture references.

**Conclusions:**

This study demonstrates the potential of using a multimodal genAI-driven approach to create CCNs in medical education. The “Shattered Slippers” case effectively enhanced student engagement and promoted knowledge retention in complex pharmacological topics. This innovative method suggests a novel direction for curriculum development that could improve learning outcomes and student satisfaction in medical education. Future research should explore the long-term retention of knowledge and the applicability of learned material in clinical settings, as well as the potential for broader implementation of this approach across various medical education contexts.

## Introduction

### Background

Student and trainee engagement is a critical factor in medical education, influencing outcomes such as academic achievement, overall well-being, satisfaction, and reduced burnout [1,2]. High levels of engagement have been linked to increased motivation and better learning experiences, as active participation encourages deeper understanding and application of complex material [3]. In contrast, traditional lecture-based learning often results in passive absorption of information, limiting student engagement and negatively affecting the ability to interact meaningfully with content [4]. To address this, we developed a cinematic clinical narrative (CCN), an interactive multimedia learning experience designed to enhance student engagement by integrating cinematic storytelling and narrative-based learning techniques. This method builds upon the principles of cinemeducation, a teaching approach that uses film to create emotional connections and foster active learning [5]. By using generative artificial intelligence (genAI) tools, we have further enhanced the learning experience and decreased the barrier to entry for instructors, making it more immersive and adaptable to current educational needs. GenAI has been recognized as a transformative tool in reshaping medical education, offering new opportunities for interactive, technology-driven learning environments that promote active student engagement [6,7].

The target audience for our CCN comprises first-year medical students learning pharmacology related to the immune system. Medical students often face a knowledge gap in understanding complex pharmacological interactions and the intricacies of immune responses largely due to the difficulty of the material [8,9]. Furthermore, there is speculated to be a skill gap in medical and other professional health science students in applying theoretical knowledge to clinical scenarios [10] and the real problem of burnout due to many factors, one of which is the large amount of knowledge required to retain in a short amount of time [11]. The CCN aims to address these issues by enhancing comprehension, clinical application skills, and empathy toward patients with autoimmune diseases.

The CCN used a unique instructional approach by merging cinemeducation [5] with multiple genAI platforms, tailored for first-year medical students in pharmacology. This method addresses the challenge of enhancing engagement and knowledge retention in complex subjects such as immune system pharmacology. Unlike traditional didactic teaching, our approach, supported by others advocating for innovative teaching strategies, uses storytelling to deepen understanding and empathy [12-14]. Use of genAI in medical training, particularly in personalizing learning experiences and competencies for genAI-based tools, is also a current area of active research [15,16]. This aligns with other researchers who highlight the importance of interactive and engaging content in medical education [17]. Our project also leverages the effectiveness of narrative-based learning, which offers an experiential learning environment over conventional teaching methods and is more accurate to real-world situations [18].

Medical students often struggle to engage with and retain complex pharmacological concepts, especially in preclinical education, where traditional teaching methods can lead to passive learning and poor knowledge retention. To address this challenge, we developed and implemented a novel instructional approach, CCNs, which leverages multimodal genAI tools to create immersive, engaging learning experiences. The aim of this study is to evaluate the effectiveness of these genAI-enhanced CCNs in increasing student engagement, interest, and knowledge retention in medical pharmacology concepts. We tested this intervention by assessing student interest using the Situational Interest Survey for Multimedia (SIS-M) and measuring examination performance on content covered by the CCNs. We hypothesize that students exposed to CCNs will report higher levels of engagement compared with traditional case-based learning and have passing examination grades on questions related to the CCN.

### Theoretical Framework

The instructional method in the CCN uses contemporary educational theories emphasizing active, learner-centered approaches. Drawing inspiration from the Constructivist Learning Theory, which advocates for knowledge construction through experience [19], our approach uses an adaptation of cinemeducation to create an immersive learning environment [5]. This also aligns with Mayer’s Cognitive Theory of Multimedia Learning, which suggests that learning is enhanced through multimodal presentations [20]. Furthermore, our multimodal use of various genAI platforms for content development is informed by the Technological Pedagogical Content Knowledge (TPACK) framework [21], ensuring an effective integration of technology in teaching. This methodology responds to identified needs in medical education for more engaging and effective teaching strategies, bridging theory and practice in a novel and impactful way.

## Methods

### Participants and CCN Design Overview

This study was conducted at the University of Idaho WWAMI Medical Education Program, which is part of a collaborative University of Washington School of Medicine program serving Washington, Wyoming, Alaska, Montana, and Idaho. The WWAMI program provides medical education to students across these states, offering them the opportunity to complete their first 2 preclinical years of medical school in their home states before transitioning to clinical training. The target learners for this study were first-year medical students in the WWAMI program enrolled in a 6-week foundational infections and immunity course, which included topics covering immune system pharmacology. Students in this course attend pharmacology lectures that culminate in clinical cases, allowing them to apply their newly acquired knowledge of medications to real-world patient scenarios.

We decided to reimagine one of these cases into “Shattered Slippers,” a CCN that was presented as a fictional sequel to the movie “Another Cinderella Story” (MultimediaAppendices 1  2). This fictional sequel features the star from the original movie, Selena Gomez, which was purposeful, given her real-life battle with lupus and her experience receiving a kidney transplant. This choice not only provides a strong thematic link connecting the CCN to the source material but also serves to humanize and demystify the conditions under study.

**Figure 1.**
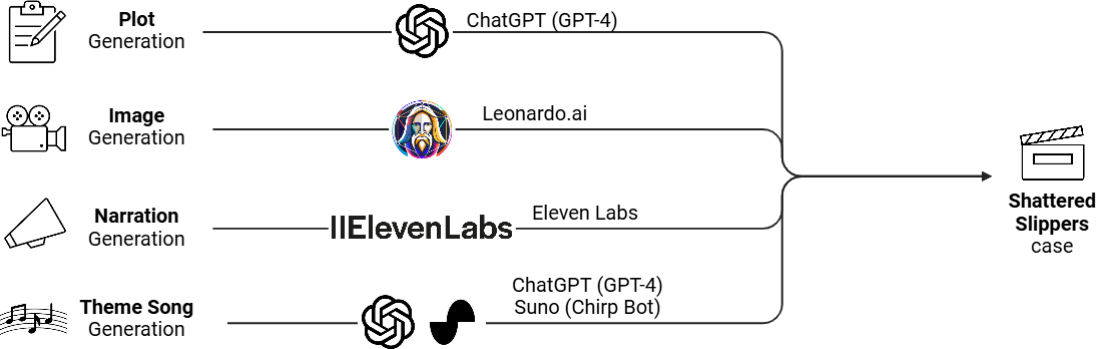
Multimodal generative artificial intelligence (genAI) case generation approach. Each portion of the case used a different genAI platform for material generation. These included ChatGPT (GPT-4), Leonardo.ai, Eleven Labs, and Suno.

The development of “Shattered Slippers” used a suite of genAI platforms to create an immersive and engaging learning experience (Figure 1). The plot was crafted using GPT-4, known for its language understanding and generation capabilities. For visual imagery, Leonardo.ai and Stable Diffusion were used to generate high-quality, contextually relevant images. Narration was produced using Eleven Labs, ensuring a coherent and captivating storytelling experience. Furthermore, the theme song, integral to setting the tone of the educational module, was composed using the combined efforts of GPT-4 and Suno.

These artificial intelligence (AI)–generated materials were all integrated into 2 PowerPoint presentations. Part I of the CCN was presented at the end of a 1-hour pharmacology lecture on immunomodulatory drugs with specific focus on nonsteroidal anti-inflammatory drugs, glucocorticoids, and innate immune system inhibitors. Part II of the CCN was presented 4 weeks later at the end of a 1-hour pharmacology lecture on immunomodulatory and transplant drugs with specific focus on cytokine inhibitors, cytotoxic drugs, and antimetabolites. Both lectures were presented in-person with >90% of students attending both lectures. The combined CCN is provided as a supplemental file (Multimedia Appendix 2).

At the conclusion of the course, students were informed about Selena Gomez’s actual medical journey. This revelation effectively bridged the gap between the fictional narrative of “Shattered Slippers” and real-world medical scenarios, thereby enhancing the educational impact and relevance of the clinical cases discussed.

### Plot Development

The process of developing the plot for “Shattered Slippers” began with a reimagining of a clinical case initially presented in the first-year medical school curriculum. This original case centered around a ballerina struggling with rheumatoid arthritis, where students were tasked with diagnosing the sources of her pain and inflammation and selecting suitable immunomodulatory medications.

**Figure 2.**
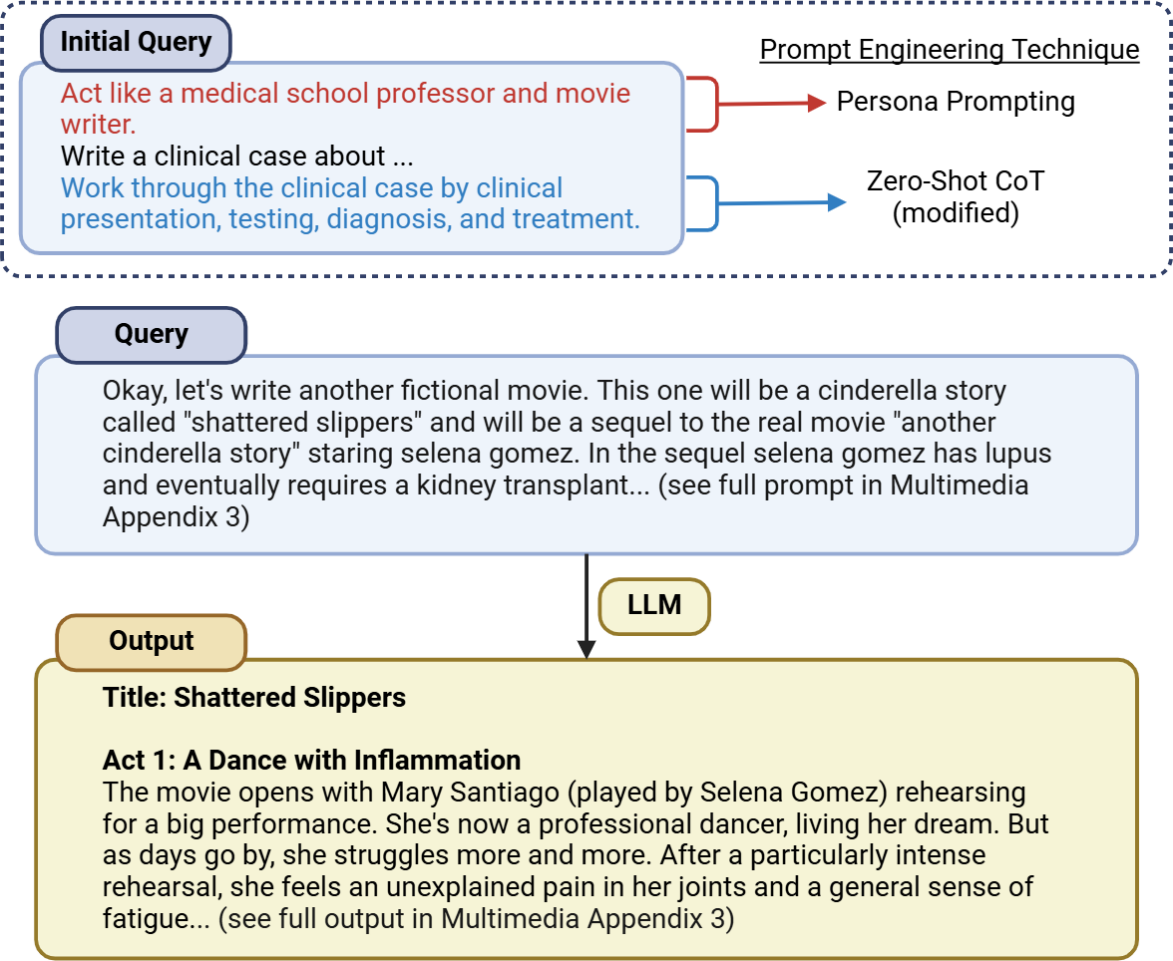
Excerpt of plot generation. The initial prompt in the conversation covered the development of a separate CCN. Prompt engineering techniques in this initial prompt included Persona Prompting [23,24] and a modified version of Zero-Shot CoT [25]. Excerpts of the first prompt and output related to the Shattered Slippers CCN are provided. CCN: cinematic clinical narrative; CoT: Chain of Thought; LLM: large language model.

Using ChatGPT (GPT-4) [22], a large language model (LLM), we transformed this clinical scenario into a compelling narrative for “Shattered Slippers.” The sequential steps of the medical case were input into GPT-4, with instructions to adapt these into a fictional storyline (Figure 2 and Multimedia Appendix 3). To enhance thematic resonance and real-world connection, the ballerina’s diagnosis in the plot was altered from rheumatoid arthritis to lupus, mirroring the real-life medical condition of Selena Gomez, who stars in the CCN.

Further expanding the scope of the narrative, the plot incorporated a kidney transplant storyline. This addition served a dual purpose. First, it aligned with the second lecture on immunoregulatory pharmacology focusing on organ transplant pharmacology. Second, it resonated with Selena Gomez’s personal medical history, as she has undergone a kidney transplant. This incorporation not only ensured continuity with the educational objectives of the course but also added depth and authenticity to the fictional narrative, making it more engaging and relatable for the students.

### Image Generation

**Figure 3.**
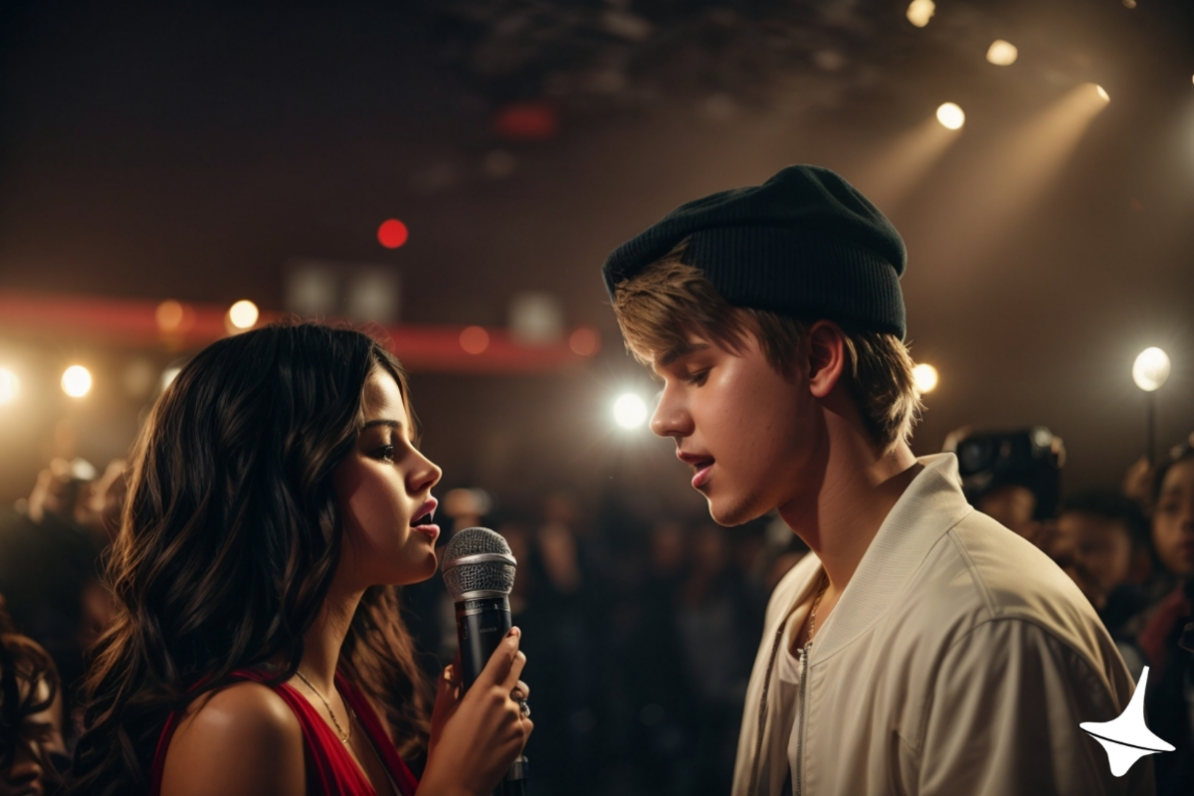
Artificial intelligence (AI)-generated image of Selena Gomez singing with Justin Bieber. The prompt used was “adult Selena Gomez and Justin Bieber singing together.” The spinning top in the bottom right corner was added as a watermark to denote an AI-generated image. Generated with Leonardo.ai.

In order to create a more immersive educational experience, fictional images were integrated into the “Shattered Slippers” case study. These images were generated using the Leonardo.ai platform [26], which harnesses the capabilities of the Stable Diffusion XL image–generating technology (Figure 3 and Multimedia Appendix 4).

In an effort to maintain transparency and distinguish between real and AI-generated content, all images depicting real people were marked with an “AI-generated image” icon. This icon, chosen for its symbolic significance, is the spinning top from the movie “Inception.” The selection of this particular icon was purposeful; it serves as a metaphor for the increasingly blurred lines between reality and artificial constructs, mirroring the movie’s thematic exploration of distinguishing reality from illusion. This concept was explained to the students prior to their engagement with the case, setting the stage for a thoughtful consideration of the role and impact of genAI in content creation. This iconography not only helped in identifying AI-generated images but also subtly underscored the advanced capabilities of genAI in creating hyperrealistic images.

### Narration Generation

Enhancing the immersive aspect of the CCN, an audio narration was incorporated to accompany the text on the PowerPoint slides. This element was designed to emulate the experience of listening to a movie narrator, thereby bringing the story of “Shattered Slippers” to life in an auditory format. To achieve this, the finalized script of the plot was submitted to the Eleven Labs platform [27], which specializes in converting text into lifelike audio narration (Multimedia Appendix 5).

Each of these audio narrations were incorporated into their corresponding PowerPoint slides. As each slide was presented during the course, the audio narration played automatically, further synchronizing the visual and auditory elements of the learning experience. This integration of audio narration with the visual content not only enriched the storytelling aspect of the module but also supported diverse learning styles, facilitating a more engaging and multisensory educational experience for the students.

### Theme Song Generation

Although not directly educational, a theme song for “Shattered Slippers” was created to complete the immersive experience. The inclusion of a theme song aimed to add an additional layer of engagement and context to the fictional movie, contributing to a more comprehensive and cinematic learning environment.

The lyrics for the theme song were generated using GPT-4 [22]. Following the lyric generation, Suno Chirp Bot, a genAI tool for music composition [28], was used to create the melody and vocals for the theme song. This genAI-driven process allowed for a harmonious blend of lyrics and music, resulting in a fully rendered theme song (Multimedia Appendix 6).

Once completed, the theme song was embedded into the PowerPoint presentation. This musical addition served as a capstone to the multisensory educational module, further enriching the student’s experience by providing a unique auditory element that complemented the visual and textual components of “Shattered Slippers.”

### Data Collection

The “Shattered Slippers” CCN was integrated into 2 distinct pharmacology lectures, both of which focused on medications used in immune system modulation. The target audience for this CCN was a class of 40 first-year medical students (n=40). This approach aimed not only to enrich their understanding of immunomodulatory pharmacology but also to engage them in a unique and memorable learning experience.

To evaluate student interest in the CCN as an educational tool, at the conclusion of the course, students were invited to participate in a feedback process using the SIS-M [29-31] (Table 1) of which 18 students responded (n=18). The SIS-M was developed by Dr Tonia Dousay, a professor in instructional design and educational technology, to assess various constructs of situational interest in multimedia-based learning environments. Originally created for the educational field, the SIS-M focuses on adult learners and measures constructs such as triggered situational interest (initial engagement with multimedia), maintained interest, and value interest (perceived usefulness of the content). The survey was originally used to evaluate the effectiveness of multimedia in promoting engagement and motivation in higher education and adult learning settings [29,30] and has recently been used in medical education research [31], making it an appropriate tool for assessing learner engagement in this study. This survey was used to capture their views and opinions on the “Shattered Slippers” case, providing insights into student engagement, interest, and the overall impact of the CCN on their learning experience. The survey includes items to rank on a 1‐5 scale (1=strongly disagree, 5=strongly agree), a question asking for preference of clinical case format, and an open-ended question asking, “Why do you think this is your preference.” The CHERRIES report for this survey is supplied (Multimedia Appendix 7).

**Table 1.** SIS items.

SIS^a^ type	Survey item
SI-triggered	The multimedia presentation was interesting.
SI-triggered	The multimedia presentation grabbed my attention.
SI-triggered	The multimedia presentation was often entertaining.
SI-triggered	The multimedia presentation was so exciting, it was easy to pay attention.
SI-maintained-feeling	What I learned in the multimedia presentation is fascinating to me.
SI-maintained-feeling	I am excited about what I learned in the multimedia presentation.
SI-maintained-feeling	I like what I learned in the multimedia presentation.
SI-maintained-feeling	I found the information in the multimedia presentation interesting.
SI-maintained-value	What I studied in the multimedia presentation is useful for me to know.
SI-maintained-value	The things I studied in the multimedia presentation are important to me.
SI-maintained-value	What I learned in the multimedia presentation can be applied to my job.
SI-maintained-value	I learned valuable things in the multimedia presentation.

a SIS: Situational Interest Survey.

### Data Analysis

The research team used Microsoft Excel for the analysis of the SIS-M survey results. The average class pharmacology examination grades (n=40) from questions covered by the “Shattered Slippers” case study (n=2) were analyzed for achievement data. These included a multiple-choice question, selected by the course lead (not the study author) from a pool of questions that tested pharmacology content covered in each pharmacology lecture. The questions were administered during the students’ weekly examinations, scheduled for the week immediately following the presentation of the material. Importantly, these questions were modeled after USMLE-style step 1 board questions, which assess students’ ability to apply their pharmacological knowledge in a clinical context. Using this format provides a rigorous and standardized measure of student understanding of the material, ensuring that the assessment reflects the type of knowledge and critical thinking required for success on future board examinations.

The SIS-M survey’s analysis focused on various dimensions of situational interest: triggered interest, maintained-value (MV), maintained interest, and maintained-feeling (MF). Thematic analysis was conducted using ChatGPT (GPT4o and o1-preview) and Claude 3.5 Sonnet. This involved generating initial codes and identifying themes, followed by the researcher combining and refining these themes for overlap and relevancy between the 3 LLMs [31]. Prompt engineering techniques used included Persona Prompting [23,24], Zero-Shot Chain of Thought (CoT) [25], and Self-Criticism [32]. The Zero-Shot Chain of Thought prompting was not used with the ChatGPT o1-preview model, as it has built-in Tree-of-Thought functionality in every output. The initial prompt was the following:

*Act like a brilliant medical education researcher. I am doing a study on a Cinematic Clinical Narrative (CCN) which is an educational tool that combines clinical case studies with storytelling techniques typically seen in movies or TV shows. By embedding medical information within a compelling fictional storyline, CCNs help medical students retain complex medical concepts in an engaging, memorable way. The CCN in the study was called “Shattered Slippers,” was a fictional sequel to the movie “Another Cinderella Story,” and stars Selena Gomez. It covered the topics of immunomodulatory medications for treating lupus, and kidney transplants. I surveyed the participants on their preference of the CCN over traditional clinical cases and asked them to explain their preference. Please perform a thematic analysis on the below participant responses marked between <response> </response>. Let’s work this out in a step by step way to be sure we have the right answer.*

<response>

*Participant responses here*

</response>

This was then followed by the following Self-Criticism prompt: “Please reflect on your previous answer for any errors.”

### Ethical Considerations

This educational research was approved as exempt by the institutional review board of the University of Idaho (21-223). As the CCN incorporated references to real celebrities and included AI-generated images of actual people, we consulted legal counsel to ensure compliance. The counsel advised that, given the educational context and the clear labeling of images as AI-generated rather than real, the usage was permissible. Furthermore, we end the CCN with a brief description of the real-life health struggles of the celebrities, which is all public information. However, since this remains a legally gray area, we recommend exercising caution in future projects that use similar techniques. The SIS-M was conducted anonymously to ensure the confidentiality of participants’ responses. No identifying information was collected, allowing students to provide honest feedback without concern for personal attribution.

## Results

The quantitative assessment of the “Shattered Slippers” CCN using the SIS-M is summarized in Table 2. The results indicated high levels in participants’ interest with the “Shattered Slippers” CCN, with the majority of students (14/18) indicating a preference for the CCN over traditionally presented clinical cases, only 1 student preferring the traditional approach, and 3 expressing no preference (Table 3).

**Table 2.** Situational Interest Survey for Multimedia results (N=18): scores.

Question	Minimum^a^	Maximum^a^	Mean^a^	SD	Variance
The Shattered Slippers case was interesting.	4.00	5.00	4.61	0.49	0.24
The Shattered Slippers case grabbed my attention.	4.00	5.00	4.72	0.45	0.20
The Shattered Slippers case was often entertaining.	3.00	5.00	4.67	0.58	0.33
The Shattered Slippers case was so exciting, it was easy to pay attention.	3.00	5.00	4.33	0.58	0.33
What I learned from the Shattered Slippers case is fascinating to me.	4.00	5.00	4.39	0.49	0.24
I am excited about what I learned from the Shattered Slippers case.	4.00	5.00	4.39	0.49	0.24
I like what I learned from the Shattered Slippers case.	3.00	5.00	4.39	0.59	0.35
I found the information from the Shattered Slippers case interesting.	4.00	5.00	4.33	0.47	0.22
What I studied in the Shattered Slippers case is useful for me to.	4.00	5.00	4.50	0.50	0.25
The things I studied in the Shattered Slippers case are important to me.	3.00	5.00	4.28	0.56	0.31
What I learned from the Shattered Slippers case can be applied to my major/career.	3.00	5.00	4.44	0.60	0.36
I learned valuable things from the Shattered Slippers case.	4.00	5.00	4.44	0.50	0.25

a Rated on a 5-point scale (1=Strongly disagree, 5=Strongly agree).

**Table 3.** Situational Interest Survey for Multimedia results (N=18): preferences for case type.

Which case type do you prefer?	Count
Traditional case studies	1
Shattered Slippers case study	14
No preference	3

Participants indicated a high average triggered situational interest in the CCN (mean 4.58, SD 0.53), as well as high maintained interest scores indicated by the students (mean 4.40, SD 0.53).

The results for MF interest indicated high MF in students receiving the CCN (mean 4.38, SD 0.51). A feeling of educational value by the participants was supported by high scores for MV interest (mean 4.42, SD 0.54).

Bridging quantitative data with qualitative insights, the survey conducted among participants also provided an open-ended question for students to reflect on their opinion of the CCN. Thematic analysis of the responses revealed the following:

- *Enhanced engagement through storytelling and entertainment*: The combination of storytelling and entertainment in the CCN heightened student engagement, making the learning process more enjoyable and effective compared with traditional methods.
- *Improved memorability and recall of medical concepts*: The CCN’s engaging narrative and multimedia elements enhanced memory retention, making complex medical information more accessible and memorable.
- *Relatability through pop culture and personal connection*: Leveraging familiar pop culture icons such as Selena Gomez helped students form a personal connection with the material, enhancing engagement and motivation to learn.
- *Preference for interactive and detailed learning*: Some students value interactive learning environments and detailed information, suggesting that while the CCN is engaging, it could be further enhanced by incorporating active learning elements and comprehensive content.
- *Suggestions for improvement*: Attention to technical elements, such as the use of genAI voice narration, could improve the overall effectiveness and reception of the CCN.

The thematic analysis reveals that the CCN “Shattered Slippers” was preferred over traditional case studies due to its engaging storytelling, enhanced memorability, and relatability through pop culture references. While students appreciated the innovative approach, some expressed a desire for more interactive learning methods and provided suggestions for technical improvements. Incorporating these insights can further refine the CCN as a valuable tool in medical education.

In addition to the survey feedback from the SIS-M, the success of the “Shattered Slippers” CCN was further demonstrated academically. Students displayed strong comprehension and knowledge of the material covered, achieving an average score of 88% on examination questions pertaining to the case study content. This high performance underscores the effectiveness of the CCN as a teaching tool, suggesting that it may also be useful in promoting academic performance as well as student preference and interest.

## Discussion

### Principal Findings

The “Shattered Slippers” CCN supports the pedagogical value of integrating innovative genAI-driven methods and culturally resonant themes into medical education. Our study shows the capacity of this approach to not only enhance student interest but also promote their understanding and retention of complex subject matter. Furthermore, it adds very little to no extra time to the lecture material, as it basically reskins the existing material into a more cinematic experience. This is particularly important, as many new active learning teaching methodologies either extend the amount of time students spend with the material or cause instructors to remove large amounts of material in order to incorporate novel active learning activities. We considered it ethical to clearly mark AI-generated images of real individuals to avoid confusion but did not deem it necessary to label AI-generated material such as text or audio that was not mimicking a real-world person. As genAI models continue to improve in generating realistic images and cloned voices, it will become increasingly important to label AI-generated materials that mimic real-world individuals to prevent confusion with reality and avoid potential legal issues.

This study shows the importance of engaging students beyond conventional didactic methods, suggesting that the inclusion of elements such as plot development, multimedia, and popular culture can make learning more relatable and impactful. The feedback from the SIS-M supports that this approach can effectively address the initial problem of student disengagement and the need for more effective educational strategies as identified in the introduction.

The process of creating CCNs with genAI tools is highly efficient and cost-effective. Designing the case outline took about a day, while plot and narration generation were completed in seconds using GPT-4 and Eleven Labs. Image and theme song generation took under an hour each, with slight delays due to iterative refinement. Overall, the time investment was minimal compared with traditional methods. The required technical skills are basic, involving familiarity with genAI platforms for text, image, and audio generation and standard project management skills to integrate these elements into a PowerPoint slide deck. In terms of cost, the only expense was a US $20 per month subscription to ChatGPT; other platforms were used on free tiers. This low cost, combined with fast production times, makes migrating to this format highly accessible and efficient for educators, offering significant time and cost savings compared with traditional content creation methods of this caliber.

Future directions of this work will explore how similar immersive educational experiences can be scaled and adapted for diverse student populations and learning environments. The versatility of genAI-enhanced CCNs extends beyond pharmacology, offering potential applications in other areas such as anatomy, pathology, and clinical skills. This pedagogical strategy can be adapted to various medical disciplines, making abstract topics more engaging and accessible to diverse learners. It also asks questions on how educational policies might evolve to integrate this type of AI-generated material into curricula systematically. As genAI becomes more integral to education, policies must address both the ethical use of genAI and the need for genAI literacy among educators and students. Personalized, genAI-driven learning experiences could revolutionize how content is delivered, providing flexibility and tailored learning opportunities. There is an opportunity to explore interdisciplinary collaborations, merging medical education with fields such as AI, storytelling, and multimedia design. These collaborations could further refine educational tools and help bridge the gap between traditional learning and modern health care technologies, fostering genAI literacy in future medical professionals. This promising pilot study shows potential for scalability and broad applicability of genAI-enhanced CCNs. The strategy offers a model for transforming how complex medical topics are taught, providing a scalable, engaging solution that can be adapted across different medical content areas to meet evolving educational needs.

### Limitations

Our project has limitations in terms of cultural adaptability due to its reliance on specific cultural references and celebrity figures, which may not resonate with all audiences. Furthermore, the use of genAI technologies presents challenges in environments with varying levels of technological resources and differing instructor familiarity with these platforms. While the skills required to effectively use genAI can vary depending on the model, these challenges are mitigated by the increasing availability of more user-friendly genAI platforms. These platforms are simplifying AI integration in educational contexts, expanding the potential for their broader application. For instance, prompt engineering, which is crucial for optimizing output from LLMs, is becoming less essential with newer versions such as ChatGPT’s o1-preview model, which incorporates many of these strategies into the system itself. This reduces the need for advanced user expertise and lowers the barrier to efficient LLM use.

Another limitation of our study is the process of validity checking for AI-generated content. Although the materials were reviewed by medical professionals, including physicians, PhDs, and PharmDs, to ensure accuracy, the use of genAI introduces potential risks in content reliability, especially as AI-generated content may produce subtle inaccuracies or lack the nuanced context that a human expert might provide. Future implementations of this approach would benefit from a formalized validation process to ensure that the clinical and educational integrity of AI-generated materials is maintained.

The evaluation methodology, focusing on immediate reactions via the SIS-M, provides a single time point of the resource’s impact but does not capture the longevity of knowledge retention or the applicability of the learned material in clinical settings. Furthermore, the study included a limited sample size, with only 18 respondents to the SIS-M survey, which may not provide a comprehensive view of the broader student population. Future research could explore longitudinal studies to measure the lasting educational benefits of such methodologies with a larger participant population.

Furthermore, our study lacked a control or comparison group, a common challenge in medical education research. All students in the study were exposed only to the CCN case, and without a traditional case-based learning comparison, it is difficult to isolate the exact impact of the CCN on student performance. While we acknowledge that a control group could provide valuable insights, the integration of such comparisons is often logistically difficult in medical school settings. Future studies could address this by designing more controlled experimental conditions or through the use of quasi-experimental designs to better understand the differential effects of various educational interventions on learning.

### Conclusions

The “Shattered Slippers” CCN demonstrates the effectiveness of combining cinemeducation with genAI in medical education. This approach enhanced student engagement, promoted knowledge retention, and offered a novel perspective on complex pharmacological clinical cases. The application and positive student feedback suggest that this multimodal genAI approach to educational content creation has potential for broader application in medical education. Our project also highlights the need for continuous innovation and adaptation in teaching methodologies to meet the evolving demands of health care education. Future research and development in this area could further transform medical education, making it more engaging, effective, and aligned with modern technological advancements.

## Supplementary material

10.2196/63865 Multimedia Appendix 1 Shattered Slippers: cinematic clinical narrative.

10.2196/63865 Multimedia Appendix 2 Shattered Slippers full presentation.

10.2196/63865 Multimedia Appendix 3 ChatGPT plot generation.

10.2196/63865 Multimedia Appendix 4 Leonardo.ai image generation.

10.2196/63865 Multimedia Appendix 5 Eleven Labs narration generation and audio clips.

10.2196/63865 Multimedia Appendix 6 ChatGPT and Suno Chirp Bot theme song generation and audio clip.

10.2196/63865 Multimedia Appendix 7 Situations Interest Survey of Multimedia CHERRIES (Checklist for Reporting Results of Internet E-Surveys) report.

## Abbreviations

AI: artificial intelligence
CCN: cinematic clinical narrative
genAI: generative artificial intelligence
LLM: large language model
MF: maintained-feeling
MV: maintained-value
SIS-M: Situational Interest Survey for Multimedia
TPACK: Technological Pedagogical Content Knowledge
