# Supplementary material for: Enhancing Medical Student Engagement Through Cinematic Clinical Narratives: Multimodal Generative AI–Based Mixed Methods Study
Source: JMIR Med Educ. 2025 Jan 6;11:e63865. doi: 10.2196/63865 (PMC11751740; doi:10.2196/63865)
Supplement: Multimedia Appendix 1 [file mededu-v11-e63865-s001.pdf]

Learning objectives

1. Describe the major classes of anti-inflammatory medications with regard to mechanism of action, pharmacological properties, clinical use and adverse effects.
2. Identify the mechanism of action, adverse effects and clinical use of medications that target components of the innate immune system (see drug list for specific agents)
3. Describe the major classes and mechanism of action of immunosuppressive agents that disrupt the adaptive immune system.
4. Explain how the mechanisms of immune-mediated injury inform the use of immunosuppressive medications in treatment of atopic disease, general autoimmunity and organ transplants.
5. Discuss the use and mechanism of antibodies as immunosuppressive therapeutic agents.
6. Predict potential adverse effects including what types of infections might be expected in a patient being treated with common immunosuppressive medications

Shattered Slippers: Act 1

Mary Santiago (played by Selena Gomez) is rehearsing for a big performance. She's now a professional dancer, living her dream. But as days go by, she struggles more and more. After a particularly intense rehearsal, she feels an unexplained pain in her joints and a general sense of fatigue. She confides in her best friend, Tami, who suggests she might just be overworking herself and recommends an over-the-counter, reversible, short-acting medication for the pain and inflammation. Trusting Tami's advice, Mary starts taking the drug daily and it seems to help.

What treatment did she most likely start taking?

Ibuprofen

Ibuprofen is a short-acting, reversible NSAID that blocks the production of inflammation inducing prostaglandins

Shattered Slippers: Act 1

She presents to your office a week later with the complaint of 2 days of fever, headache, sore throat as well as a muffled sounding voice. You perform a physical exam and notice that her tonsils are swollen with exudate, palatal petechiae, and swollen anterior lymph nodes. You perform a throat swab for a rapid antigen test and back up cultures are sent.

What is responsible for the observed symptoms?

Bacterial infection

Symptoms such as fever, and sore throat due to swollen tonsils with exudate and lymph nodes suggest a bacterial infection.

Shattered Slippers: Act 1

Lab results return Group A Streptococcus infection, and you treat her with penicillin.

Note: When asked if she was on any medications, she responded with "No."

How would her OTC ibuprofen likely affect her symptoms?

Decrease swelling

NSAIDs are anti-inflammatory and would most likely help her symptoms by decreasing throat swelling.

Shattered Slippers: Act 1

The antibiotic treatment clears the infection and she returns back to life as normal. With her next big performance coming up, rehearsals start to intensify and so does her pain. Her joints, particularly her wrists and knees, start to ache after prolonged dance sessions. The ibuprofen, which initially seemed effective, doesn't offer the same relief anymore. Tami notices Mary wincing during certain dance moves and urges her to see a doctor. At the doctor's office, after a thorough examination, the physician voices concerns about possible inflammation and prescribes a long-acting NSAID to provide sustained relief.

What treatment was prescribed?

Naproxen

NSAID similar to ibuprofen but with a longer $t_{1/2}$

Shattered Slippers: Act 1

Mary stops taking the ibuprofen and starts on naproxen which provides an initial improvement. Her dance performance gets a boost, and she feels hopeful. However, the relief is again short-lived. As the days pass, Mary's joint pain becomes more persistent, and she starts to experience fatigue, making it hard for her to keep up with her dance routines. She starts taking the ibuprofen along with the naproxen to try and keep the pain and inflammation at bay so that she can continue dancing. Tami and her other friends begin to notice Mary's struggles. They rally around her, supporting her during rehearsals and urging her to seek further medical advice. Back at the doctor, Mary confesses to the ibuprofen. Alarmed, the doctor switches her medication to prednisone and tells her to stop taking the ibuprofen and naproxen.

What adverse effects should you warn her about with the prednisone?

Metabolic symptoms, thinning skin/easy bruising, osteoporosis

Shattered Slippers: Act 1

The doctor notices that Mary has a facial rash on her cheeks and the bridge of her nose. He runs the following tests:

- Rheumatoid factor: negative *(not rheumatoid arthritis)*
- ANA (anti-nuclear antibody): positive *(SLE)*
- *Urine: proteinuria (lupus nephritis)*

Along with SLE, did anything else likely contribute to her kidney failure?

Yes, chronic NSAIDs

One side effect of NSAIDs is kidney damage due to NSAIDs blocking vasodilation of the afferent arteriole which decreases kidney perfusion.

Shattered Slippers: Act 1

With the new diagnosis of SLE and lupus nephritis, the doctor switches Mary to hydroxychloroquine and orders more kidney function tests. Unfortunately, both of Mary's kidneys are failing and they put her on the transplant list.

Shattered Slippers will continue near the end of I&I…

Shattered Slippers: Act 2

Previously in Shattered Slippers:

Mary Santiago (played by Selena Gomez) is a professional dancer that has recently been diagnosed with systemic lupus erythematous (SLE) and lupus nephritis. She is being treated with hydroxychloroquine for her SLE and has been put on the kidney transplant list.

Shattered Slippers: Act 2

A few months have passed since Mary's diagnosis. The hospital's emergency department doors burst open, and Mary is rushed in, delirious and burning up with fever. Her mother is beside herself with worry. As the attending physician in the ED, you quickly assess Mary's condition and realize this is a severe flare-up of her SLE. Knowing the urgency, you initiate IV glucocorticoids and another medication to halt antibody production, aiming to suppress her overactive immune system.

What treatment did she most likely start taking?

Methylprednisolone + Rituximab

Methylprednisolone is an active IV glucocorticoid.

Rituximab is an anti-CD20 antibody that inhibits and depletes B-cells, thus preventing antibody production.

Shattered Slippers: Act 2

As Mary stabilizes and is moved to a regular ward, she meets a fellow patient, a young musician diagnosed with Lyme disease (played by Justin Bieber). They quickly bond over shared experiences of illness, dreams that seem just out of reach, and the medications that have become a central part of their lives.

The musician talks about his 30S inhibitor antibiotic, mentioning its quirky side effects: potential teeth discoloration and heightened sensitivity to sunlight. Mary chuckles, realizing that even in their shared challenges, there's room for humor and connection.

What medication is he taking?

Doxycycline (tetracycline)

Doxycycline is a reversible 30S inhibitor that is used to treat Lyme disease and can get incorporated into teeth and cause photosensitivity.

Shattered Slippers: Act 2

One quiet evening, the musician plays "Shattered Slippers" for Mary. The soulful tune resonates with her, symbolizing the fragility of dreams but also the undying hope of rebuilding. Together, they sing, their voices echoing through the hospital corridors, bringing a momentary respite from the usual sounds of beeping monitors and hushed conversations.

Shattered Slippers: Act 2

With her condition improving, Mary often finds herself reminiscing about her dancing days. On one such day, the musician suggests they collaborate, blending her graceful dance with his heartfelt music. They begin practicing in the hospital's courtyard, their artistry symbolizing the resilience of the human spirit.

During this period, Tami, driven by a deep bond of friendship, gets tested and discovers she's a kidney match for Mary. The news brings a ray of hope. Before the surgery, Mary is put on induction therapy that includes a drug that prevents T-cell expansion, preparing her body to accept the new kidney.

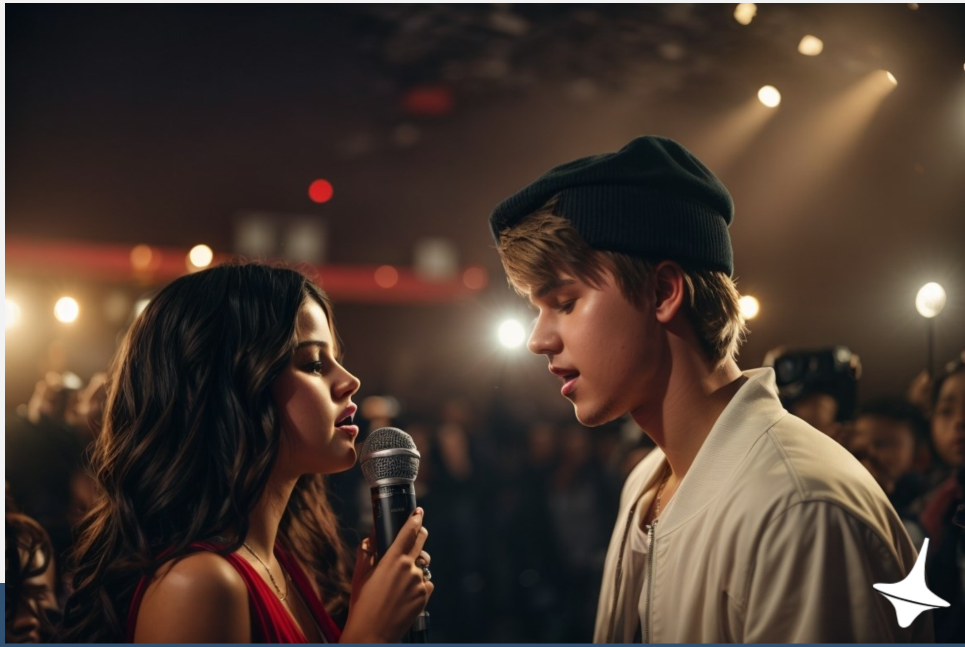

What induction therapy regimen was she put on?

Methylprednisolone + basiliximab

Methylprednisolone is an active IV glucocorticoid.

Basiliximab is an anti-IL2 antibody that prevents T-cell proliferation and differentiation.

Shattered Slippers: Act 2

The day of the surgery arrives. Tami's selfless act of donating a kidney is a testament to the depth of their friendship. The surgery is a success, a moment of triumph in Mary's battle against SLE.

As Mary starts her road to recovery, her medication regimen shifts to maintenance therapy. Her doctor performs regular kidney function tests, not only to check for rejection but also because one of the side effects of the immunosuppression therapy is nephrotoxicity.

What maintenance therapy regimen was she put on?

Prednisone + azathioprine + cyclosporine

Azathioprine was chosen over mycophenolate because she is a female of childbearing age, and cyclosporine is nephrotoxic.

Shattered Slippers: Act 2

After overcoming illness and finding harmony in music, Mary's journey is on an uplifting note. But as life's symphony plays on, new challenges await, testing her resilience and spirit once more...

Shattered Slippers will conclude in MBB...

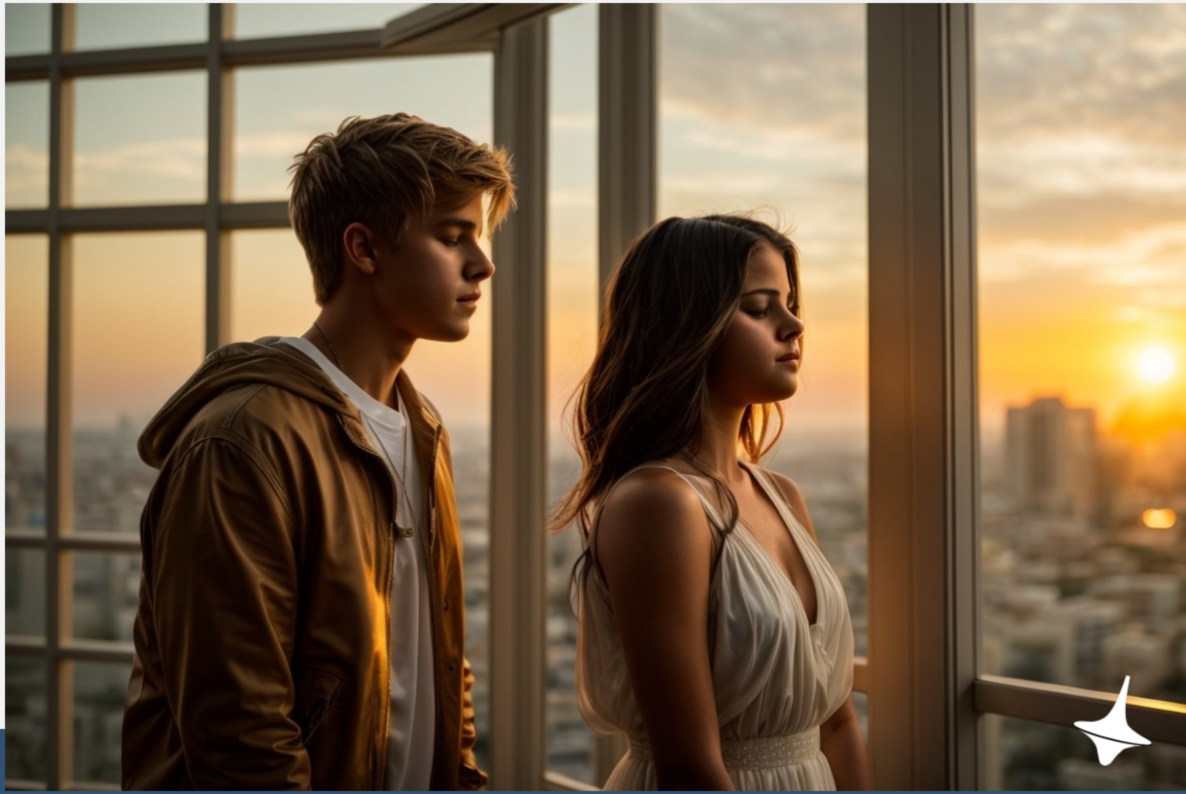

Shattered Slippers

The actress who plays the Cinderella character in the movie Another Cinderella Story is Selena Gomez. In reality, she suffers from lupus and has received a kidney transplant due to complications with this disease. She opens up about her struggles with lupus and her mental well-being in the documentary Selena Gomez: My Mind & Me on Apple TV+.

Questions?

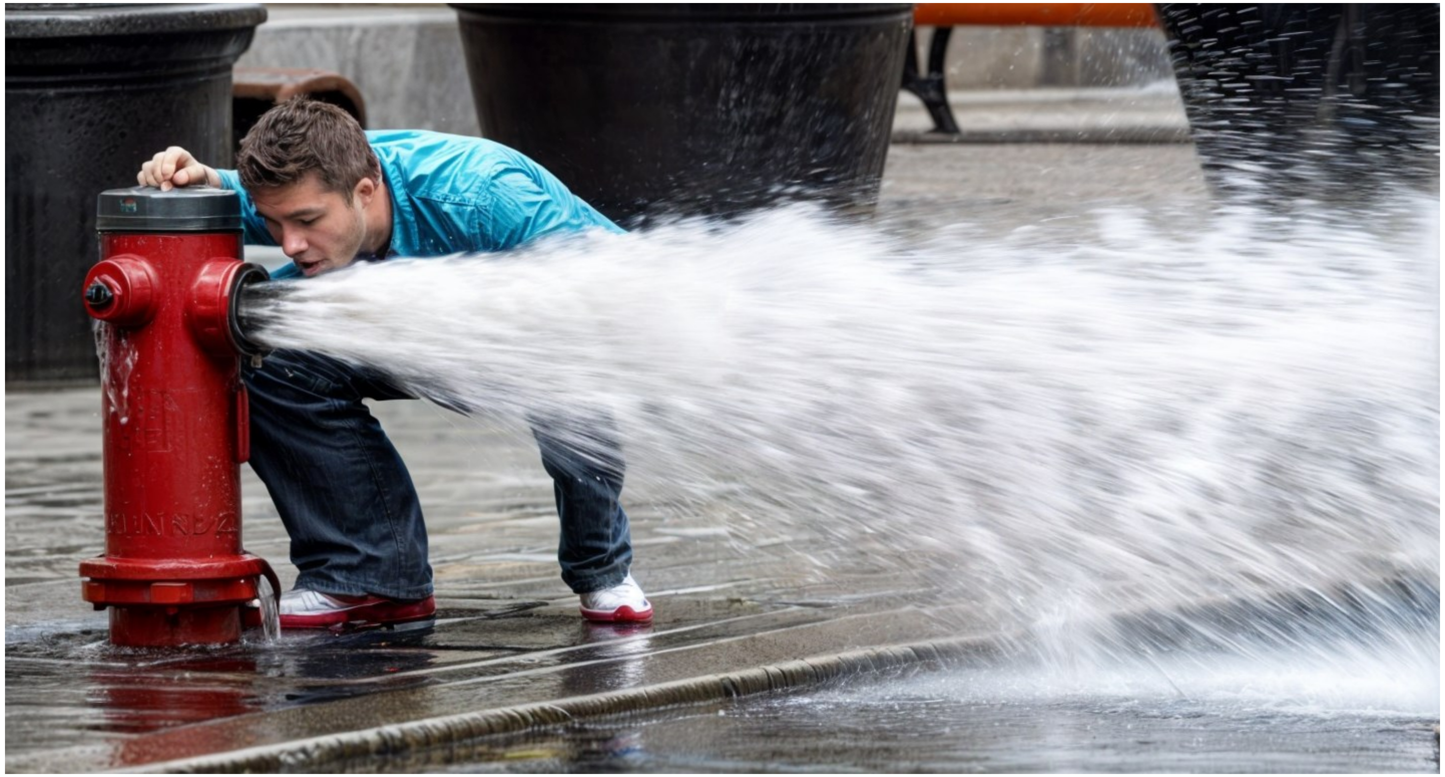

High-yield slides can be viewed at www.blandpharm.com
