# Supplementary material for: Enhancing Medical Student Engagement Through Cinematic Clinical Narratives: Multimodal Generative AI–Based Mixed Methods Study
Source: JMIR Med Educ. 2025 Jan 6;11:e63865. doi: 10.2196/63865 (PMC11751740; doi:10.2196/63865)
Supplement: Multimedia Appendix 3 [file mededu-v11-e63865-s003.docx]

**Multimedia Appendix 3. Plot Generation (GPT-4)**

| *Initial prompt in the conversation* | Act like a medical school professor and movie writer. Write a clinical case about Parkinson Disease that is based in the Back to the Future universe. The basis of the case will be that Marty and Doc go to the future and discover that future Marty has Parkinson Disease. Don't disclose that Marty has Parkinson until the end. Work through the clinical case by clinical presentation, testing, diagnosis, and treatment. |
| --- | --- |
|  |  |
| *Prompt:* | Okay, let's write another fictional movie. This one will be a cinderella story called "shattered slippers" and will be a sequel to the real movie "another cinderella story" staring selena gomez. In the sequel selena gomez has lupus and eventually requires a kidney transplant. This movie should focus on the following treatments for the first part of the movie that occurs before the lupus diagnosis and kidney transplant: NSAIDs, glucocorticoids. She should first be treated with a short term (ibuprofen) then long term (naproxen) NSAID. Then her meds should be increased to prednisone. The first part of the movie will end without knowing what inflammatory disease she has. The first part should include recognizing inflammation and treating said inflammation. The second part of the movie will include more characteristic traits of lupus (butterfly rash, more extensive joint pain, and eventual kidney failure). After diagnosis, she should be treated with hydroxychloroquine. It should also include a scene where she is rushed to the hospital by her mother because her lupus is so severe and has to be treated with methylprednisolone and mycophenolate. Then she discovers that she has kidney failure due to her lupus and requires a kidney transplant. She is started on transplant induction therapy with methylprednisolone and basilizimab and then after surgery she is moved over to maintenance therapy with prednisone, azathioprine, and cyclosporine. |
| *Output:* | **Title: Shattered Slippers**  **Act 1: A Dance with Inflammation**  The movie opens with Mary Santiago (played by Selena Gomez) rehearsing for a big performance. She's now a professional dancer, living her dream. But as days go by, she struggles more and more. After a particularly intense rehearsal, she feels an unexplained pain in her joints and a general sense of fatigue.  Concerned, her mother takes her to a doctor who prescribes ibuprofen to help with the inflammation and pain. However, the relief is temporary. Over time, her symptoms intensify, and she's prescribed naproxen, a longer-acting NSAID. But even with these medications, her health doesn’t seem to improve.  Mary's dance performances suffer, and she faces criticism from her dance director and peers. They don't understand the invisible pain she's going through. She's then started on prednisone, a glucocorticoid, to help manage the increasing inflammation.  Act 1 closes with a poignant scene where Mary, looking at her reflection in a mirror, breaks down. The audience is left in suspense, with the looming question: What is wrong with Mary?  **Act 2: Unraveling the Mystery**  Mary’s symptoms become more pronounced. She wakes up one morning with a butterfly-shaped rash on her face, and her joint pain has now become more extensive. She's unable to dance, and her world seems to be falling apart.  Her mother, noticing her declining health, rushes her to the hospital. The doctors conduct a series of tests, and she's diagnosed with lupus. To manage her severe symptoms, she's treated with intravenous methylprednisolone and started on mycophenolate.  While in the hospital, Mary bonds with another patient, a young musician with a chronic illness. They share stories, dreams, and fears. He introduces her to a song called "Shattered Slippers," symbolizing her broken dreams but also the hope to rebuild.  Mary is discharged with a prescription for hydroxychloroquine to manage her lupus symptoms. However, her journey takes another devastating turn when she discovers she has kidney failure due to her lupus. She's heartbroken but determined to fight.  **Act 3: The Gift of Life**  Mary is informed that she requires a kidney transplant. The news is overwhelming, and the waiting game begins. To prepare her for the transplant, she's started on induction therapy with methylprednisolone and basiliximab.  While waiting for a match, Mary reconnects with her past, revisiting her dance studio and reminiscing about her journey. The musician friend visits her and they collaborate on a performance – blending dance and music, showcasing their combined resilience.  News arrives that a kidney donor has been found. Mary undergoes a successful transplant surgery. After surgery, she's moved to maintenance therapy with prednisone, azathioprine, and cyclosporine to ensure the success of the transplant.  **Epilogue: Dance of Hope**  The movie concludes with Mary, post-recovery, performing at a fundraiser for lupus research and organ donation awareness. The final scene is her emotional dance performance to the song "Shattered Slippers." It's a tribute to her journey, her struggles, and her unwavering spirit. The audience gives her a standing ovation, not just for her performance, but for the battles she's fought and won.  The end credits roll with information about lupus, its symptoms, treatment, and the importance of organ donation, turning the movie into an awareness campaign. |
| *Prompt:* | Please expand the section between being treated with ibuprofen and being prescribed naproxen. |
| *Output:* | **Expanded Act 1: A Dance with Discomfort**  The movie continues with Mary Santiago preparing for her next big performance. The excitement in the dance studio is palpable. However, as rehearsals intensify, Mary begins to notice something amiss. Her joints, particularly her wrists and knees, start to ache after prolonged dance sessions. Initially dismissing it as mere fatigue, she attempts to push through.  One evening after rehearsal, Mary's pain becomes particularly bothersome. She confides in her best friend, Tami, who suggests she might just be overworking herself and recommends over-the-counter ibuprofen for the pain. Trusting Tami's advice, Mary starts taking ibuprofen, and for a few days, it seems to help.  However, over the next couple of weeks, the pain starts creeping in more frequently, even on days she doesn't rehearse. The ibuprofen, which initially seemed effective, doesn't offer the same relief anymore. Tami notices Mary wincing during certain dance moves and urges her to see a doctor.  At the doctor's office, after a thorough examination, the physician voices concerns about possible inflammation. He advises Mary to switch from ibuprofen to a longer-acting NSAID, naproxen, which might provide more sustained relief. Mary is hesitant, unsure of what's causing her pain and if this new medication would indeed help. The doctor assures her that it's a safe next step, but he also schedules some blood tests to get a clearer picture of what might be causing her symptoms.  Mary starts on naproxen and initially feels an improvement. Her dance performance gets a boost, and she feels hopeful. However, the relief is again short-lived. As the days pass, Mary's joint pain becomes more persistent, and she starts to experience fatigue, making it hard for her to keep up with her dance routines.  Tami and her other friends begin to notice Mary's struggles. They rally around her, supporting her during rehearsals and urging her to seek further medical advice. The act progresses with Mary's health seemingly in a downward spiral, leading her to be prescribed prednisone as her condition deteriorates.  Act 1 continues with Mary grappling with the unknown, her dreams of dancing at risk, and the looming question: What is happening to her body? |
| *Prompt:* | Going back to the shattered slippers movie, can you please change act 2 to contain the following: 1. Some months later, you are working in the ED and Mary is rushed in by her mother. Mary is febrile and delirious. You determine that this is a severe flare-up of her SLE and needs to be treated with strong immunosuppressive therapy. You start her on an IV glucocorticoids and a medication that prevents antibody production. 2. While in the hospital, Mary bonds with another patient, a young musician with Lyme disease (played by Justin Bieber). They share stories, dreams, and fears. He introduces her to a song called "Shattered Slippers," symbolizing her broken dreams but also the hope to rebuild. They bond by singing and comparing medications, of which he states that he is on a 30S inhibitor antibiotic that could turn his teeth black and make him sensitive to the sun. 3. While waiting for a match, Mary reconnects with her past, revisiting her dance studio and reminiscing about her journey. The musician friend visits her and they collaborate on a performance – blending dance and music, showcasing their combined resilience. 4. During this time, Tami gets tested and it turns out she is a match. Mary is put on regular induction therapy before the surgery: (methylpredisolon) and a drug that depletes T-cells (rATG). Tami donates a kidney to Mary and the surgery is successful. 5. Mary is then moved to maintenance therapy: prednisone, azathioprine (because she is of child bearing age and is thinking of starting a family with the young musician), and cyclosporine. Please rewrite sections to make the plot flow. |
| *Output:* | **Revised Act 2: From Despair to Hope**  **The Flare-Up**  A few months have passed since Mary's diagnosis. The hospital's emergency department doors burst open, and Mary is rushed in, delirious and burning up with fever. Her mother is beside herself with worry. As the attending physician in the ED, you quickly assess Mary's condition and realize this is a severe flare-up of her SLE. Knowing the urgency, you initiate IV glucocorticoids and another medication to halt antibody production, aiming to suppress her overactive immune system.  **A Melody in the Midst of Misery**  As Mary stabilizes and is moved to a regular ward, she meets a fellow patient, a young musician diagnosed with Lyme disease. He's played by Justin Bieber. They quickly bond over shared experiences of illness, dreams that seem just out of reach, and the medications that have become a central part of their lives.  The musician talks about his 30S inhibitor antibiotic, mentioning its quirky side effects: potential teeth discoloration and heightened sensitivity to sunlight. Mary chuckles, realizing that even in their shared challenges, there's room for humor and connection.  One quiet evening, the musician plays "Shattered Slippers" for Mary. The soulful tune resonates with her, symbolizing the fragility of dreams but also the undying hope of rebuilding. Together, they sing, their voices echoing through the hospital corridors, bringing a momentary respite from the usual sounds of beeping monitors and hushed conversations.  **Dancing with Destiny**  With her condition improving, Mary often finds herself reminiscing about her dancing days. On one such day, the musician suggests they collaborate, blending her graceful dance with his heartfelt music. They begin practicing in the hospital's courtyard, their artistry symbolizing the resilience of the human spirit.  During this period, Tami, driven by a deep bond of friendship, gets tested and discovers she's a kidney match for Mary. The news brings a ray of hope. Before the surgery, Mary is put on induction therapy with methylprednisolone and rATG, a drug that depletes T-cells, preparing her body to accept the new kidney.  **The Gift of Friendship**  The day of the surgery arrives. Tami's selfless act of donating a kidney is a testament to the depth of their friendship. The surgery is a success, a moment of triumph in Mary's battle against SLE.  As Mary starts her road to recovery, her medication regimen shifts to maintenance therapy. She's prescribed prednisone, azathioprine (given her desires of potentially starting a family with the musician), and cyclosporine.  **Epilogue: A New Beginning**  The film gently fades with a scene of Mary and the musician, hand in hand, looking out of the hospital window. The sun sets, casting a golden hue, symbolizing the end of a challenging chapter and the promise of a new dawn. Their journey reflects the power of human connection, the strength of friendship, and the undying spirit of hope. |
