# Supplementary material for: Enhancing Medical Student Engagement Through Cinematic Clinical Narratives: Multimodal Generative AI–Based Mixed Methods Study
Source: JMIR Med Educ. 2025 Jan 6;11:e63865. doi: 10.2196/63865 (PMC11751740; doi:10.2196/63865)
Supplement: Multimedia Appendix 4 [file mededu-v11-e63865-s004.docx]

**Multimedia Appendix 4. Image Generation (GPT-4/Leonardo.ai)**

| **Image 1** | |
| --- | --- |
| *Prompt:* | adult Selena gomez and justin bieber singing together |
| *Workflow:* | PhotoReal |
| *Pipeline:* | Alchemy |
| *Preset:* | Cinematic |
| *PhotoReal:* | On |
| *Seed:* | 267851264 |
| *Image:* | 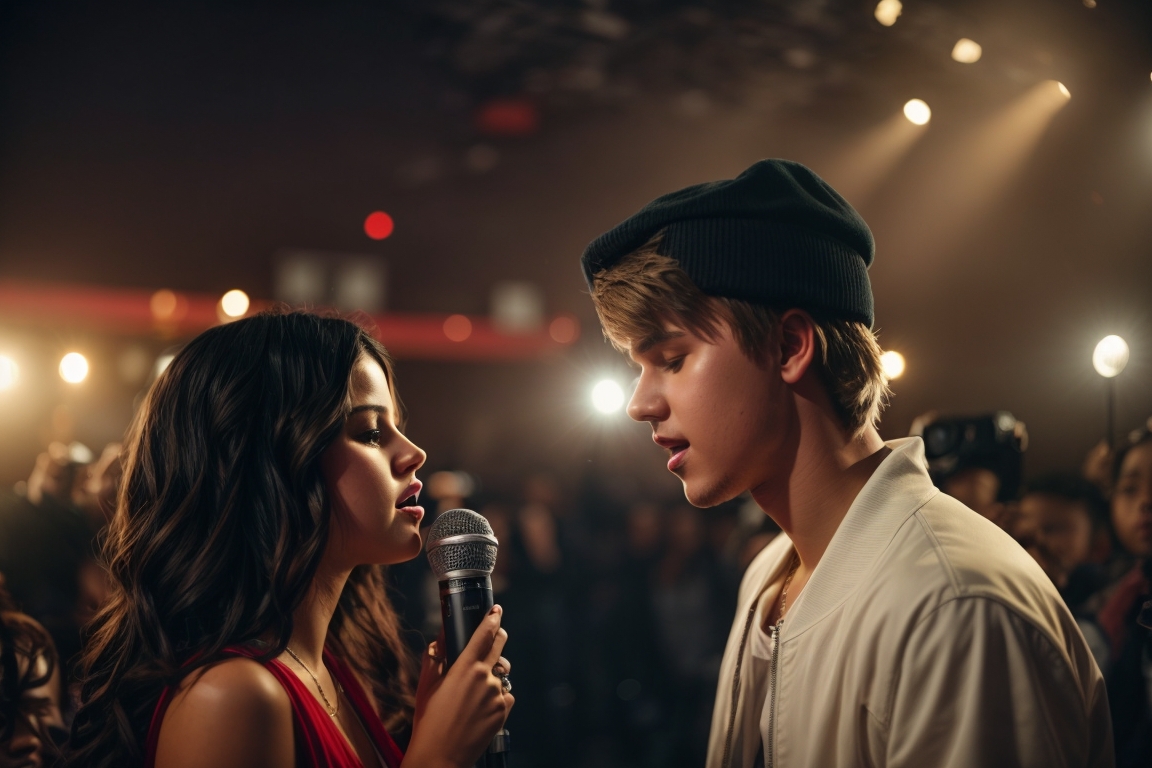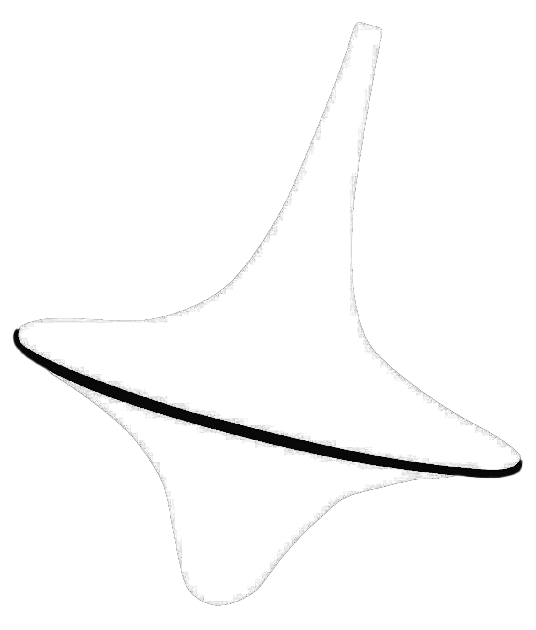 |
| **Image 2 – Final Scene** | |
| *Prompt:* | GPT-4 produced prompt (see below) |
| *Workflow:* | PhotoReal |
| *Pipeline:* | Alchemy |
| *Preset:* | Cinematic |
| *PhotoReal:* | On |
| *Seed:* | 526504704 |
| *Image:* | 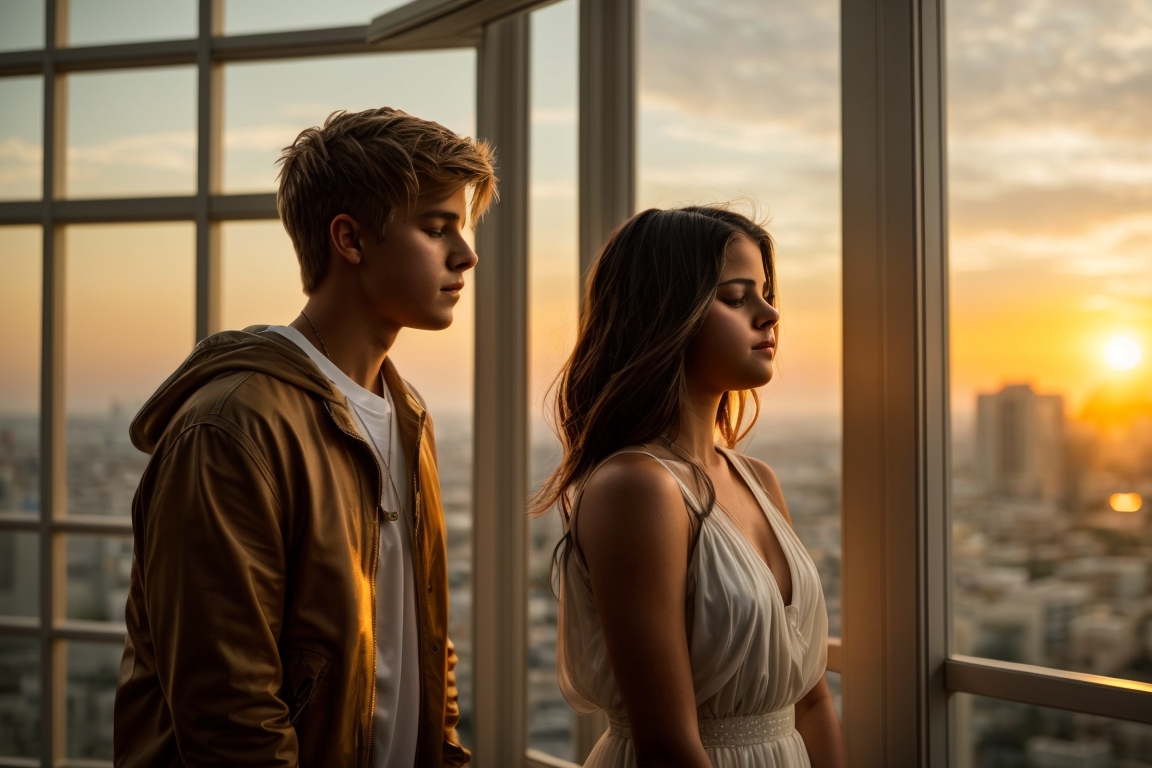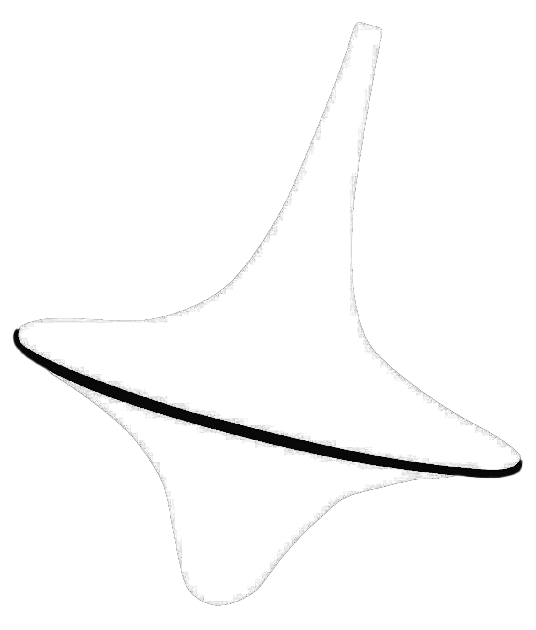 |
| *Prompt: (GPT-4)* | Please write a stable diffusion prompt that would produce an image the represents the epilogue: a new beginning. Use selena gomez instead of mary and justin bieber instead of the musician. |
| *Output:*  *(GPT-4)* | Create a serene and hopeful image capturing the essence of a new beginning. Picture Selena Gomez and Justin Bieber standing side by side, looking out of a vast hospital window. The window frames a breathtaking sunset, with the sun casting golden hues across the horizon, suggesting the close of a challenging day and the promise of a brighter tomorrow. Selena and Justin's expressions are a mix of contemplation and hope. They hold hands, symbolizing their shared journey and mutual support. The ambiance should evoke feelings of resilience, recovery, and the power of human connection. |
