# Supplementary material for: Enhancing Medical Student Engagement Through Cinematic Clinical Narratives: Multimodal Generative AI–Based Mixed Methods Study
Source: JMIR Med Educ. 2025 Jan 6;11:e63865. doi: 10.2196/63865 (PMC11751740; doi:10.2196/63865)
Supplement: Multimedia Appendix 5 [file mededu-v11-e63865-s005.docx]

**Multimedia Appendix 5. Narration Generation (Eleven Labs)**

| *Voice:* | Matilda |
| --- | --- |
| *Workflow:* | The final edit of the plot for each PowerPoint slide was entered into Eleven Labs and converted into audio narration |
