# Supplementary material for: Enhancing Medical Student Engagement Through Cinematic Clinical Narratives: Multimodal Generative AI–Based Mixed Methods Study
Source: JMIR Med Educ. 2025 Jan 6;11:e63865. doi: 10.2196/63865 (PMC11751740; doi:10.2196/63865)
Supplement: Multimedia Appendix 6 [file mededu-v11-e63865-s006.docx]

**Multimedia Appendix 6. Theme Song Generation (GPT-4/Suno)**

| **Lyric generation (GPT-4)** | |
| --- | --- |
| *Prompt:* | Please write the lyrics to the shattered slippers song that the musician sings in the story. Make it very brief. Also say what type of music it is. |
| *Output:* | **Type of Music:** Acoustic ballad with soft piano accompaniment and subtle violin undertones.  **"Shattered Slippers"**  *Verse 1:*  In a world of endless dance and grace,  I once twirled, lost in the embrace.  But now I stand, with dreams amiss,  My journey marked by a fragile twist.  *Chorus:*  Shattered slippers on the floor,  Yet I dream to dance once more.  Broken, yes, but not defeated,  With every step, life's song repeated.  *Verse 2:*  Darkness falls, yet hope remains,  In silent nights, amidst the pains.  For every crack and tear that's shown,  A story of strength, a path unknown.  *Chorus:*  Shattered slippers, dreams astray,  Yet with hope, I'll find my way.  Though they're broken, my spirit's free,  For the dance lives on inside of me.  *Outro:*  So here I stand, dreams in my grip,  With shattered slippers, I'll still trip  and twirl and leap, come what may,  For dreams reborn will light my way. |
| **Music/vocal generation (Suno Chirp Bot)** | |
| *Style:* | Acoustic guitar |
| *Workflow:* | Two sections of the lyrics were generated at a time. Each generation produced two variations, of which one was selected to continue with. At the conclusion, all three sections of the song were combined into a single track. |
