# Supplementary material for: Enhancing Medical Student Engagement Through Cinematic Clinical Narratives: Multimodal Generative AI–Based Mixed Methods Study
Source: JMIR Med Educ. 2025 Jan 6;11:e63865. doi: 10.2196/63865 (PMC11751740; doi:10.2196/63865)
Supplement: Multimedia Appendix 7 [file mededu-v11-e63865-s007.docx]

**CHERRIES Compliance for the Online Survey**

**1. Survey Design**

The online survey used in this study was designed using the Situational Interest Survey for Multimedia (SIS-M), which has been validated in previous studies on educational interventions [26-27]. The survey was conducted entirely online to assess medical students’ interest, engagement, and preference for AI-enhanced Cinematic Clinical Narratives (CCNs) versus traditional case-based learning.

**2. IRB Approval and Informed Consent Process**

The study received exemption approval from the Institutional Review Board (IRB) of University of Idaho prior to data collection. Informed consent was obtained from all participants before the survey began. Participants were provided with a clear explanation of the study’s purpose, expected duration, anonymity, and the voluntary nature of participation. Consent was confirmed through a required check-box at the beginning of the survey.

**3. Data Protection and Privacy**

Participant anonymity was maintained by not collecting any identifiable personal information, including names, IP addresses, or email addresses. Cookies were not used for tracking participants. No personal data beyond the survey responses were recorded, ensuring that the data remained anonymous.

**4. Development and Pretesting**

The online survey used in this study was designed using the Situational Interest Survey for Multimedia (SIS-M), which has been developed and pretested in previous studies on educational interventions [26-27].

**5. Recruitment Process**

Participants were recruited from a cohort of first-year medical students enrolled in the University of Washington School of Medicine attending at the University of Idaho Site. A recruitment email containing a link to the survey was sent to the entire cohort (n=40). The email explained the study's goals, the voluntary nature of participation, and the expected completion time of 5-10 minutes.

**6. Survey Administration**

The survey was administered using Qualtrics to ensure accessibility across devices, including laptops, tablets, and smartphones. The survey was accessible for a period of two weeks, with two reminder emails sent to increase participation.

**7. Handling of Incomplete Responses**

Incomplete surveys were excluded from the final analysis. Participants who did not complete at least 80% of the survey were not included in the data analysis. Of the 40 students invited to participate, 18 completed the survey in full (n=18).

**8. Response Rate**

A total of 40 medical students were invited to participate, with 18 completing the survey, resulting in a response rate of 45%. Partial responses were excluded from the final dataset.

**9. Survey Format and Display**

The survey was presented across multiple screens, with each question presented on a separate page to avoid overwhelming respondents. A progress bar was displayed to inform participants of their survey completion status. The questions were presented in a fixed order, as randomization was not applicable to this study.

**10. Prevention of Multiple Entries**

To prevent multiple entries from the same individual, participants were instructed to complete the survey only once. The survey platform automatically prevented duplicate entries based on device cookies.

**11. Incentives**

No incentives were provided for participating in the survey.

**12. Statistical Analysis**

Descriptive statistics were generated for each survey item, including means, standard deviations, and response frequencies. Student preferences for the CCN versus traditional case-based learning were evaluated through qualitative and quantitative analysis. The data were exported into Microsoft Excel for further analysis.
